# Assessment of predation risk through referential communication in incubating birds

**DOI:** 10.1038/srep10239

**Published:** 2015-05-18

**Authors:** Toshitaka N. Suzuki

**Affiliations:** 1 Department of Evolutionary Studies of Biosystems, The Graduate University for Advanced Studies, Kanagawa, Japan

## Abstract

Parents of many bird species produce alarm calls when they approach and deter a nest predator in order to defend their offspring. Alarm calls have been shown to warn nestlings about predatory threats, but parents also face a similar risk of predation when incubating eggs in their nests. Here, I show that incubating female Japanese great tits, *Parus minor*, assess predation risk by conspecific alarm calls given outside the nest cavity. Tits produce acoustically discrete alarm calls for different nest predators: “jar” calls for snakes and “chicka” calls for other predators such as crows and martens. Playback experiments revealed that incubating females responded to “jar” calls by leaving their nest, whereas they responded to “chicka” calls by looking out of the nest entrance. Since snakes invade the nest cavity, escaping from the nest helps females avoid snake predation. In contrast, “chicka” calls are used for a variety of predator types, and therefore, looking out of the nest entrance helps females gather information about the type and location of approaching predators. These results show that incubating females derive information about predator type from different types of alarm calls, providing a novel example of functionally referential communication.

Predation is a significant and unpredictable event that reduces the breeding success of most birds^1^,^2^, many of which produce alarm calls in response to approaching nest predators in an attempt to defend their offspring^3^,^4^. Previous research has primarily focused on parent-nestling communication in which parent birds use alarm calls when predators are present to communicate this threat to nestlings^4^. Nestlings typically respond to alarm calls by suppressing their begging calls^5^,^6^,^7^, and this prevents predators from detecting the exact location of the nest^8^. In several bird species, the acoustic structure of alarm calls varies with the type of nest predator^9^,^10^, and this variation may be used to elicit different anti-predator behaviours in nestlings^11^. These calls are considered functionally referential signals^12^,^13^, because they convey information about predator type to conspecific receivers.

Functionally referential communication may also be advantageous for breeding parents during the egg-laying and incubation stages. For example, yellow warblers, *Dendroica petechia*, produce “seet” calls when they detect a brood-parasitic cowbird during the egg-laying period^14^, and females react with a unique response that prevents brood parasitism, i.e., they rush back to and sit tightly on their nests^15^. Since other types of alarm calls do not elicit such nest protection behaviours in females^14^,^15^, the “seet” calls are considered functionally referential signals that warn females of the presence of a cowbird. During incubation periods, alarm calls are commonly produced in response to nest predators that prey on eggs^9^,^16^, but it is not clear whether these calls provide specific information about predator type to parents.

If nests face multiple nest predators that differ in their ability to access and prey on eggs, selection may favour incubating parents that use distinct anti-predator behaviours to defend their eggs. While nest predators mainly prey on eggs during the incubation stage, they often attack and prey on incubating adults that stay in their nests^17^,^18^,^19^. Therefore, nest predators may also select for different avoidance behaviours in incubating birds. One species whose nests face a variety of predators is the Japanese great tit, *Parus minor*. The tits are socially monogamous passerines that nest in cavities, and only females incubate eggs. In contrast to open-nesting species, the tits are limited by their visual fields while incubating their eggs inside their nest cavities. Males often produce alarm calls in response to nest predators during the incubation stage^9^,^16^, which may allow the incubating females to use alarm calls to assess the type of nest predator outside the nest cavities.

Previous studies have shown that Japanese great tits produce functionally referential alarm calls for different nest predators, i.e., “jar” calls for Japanese rat snakes, *Elaphe climacophora*, and “chicka” calls for other predators such as jungle crows, *Corvus macrorhynchos*, and Japanese martens, *Martes melampus*^10^,^11^,^20^ (Fig. 1). Incubating females may show distinct anti-predator responses to the two types of alarm calls, since snakes and other predators use different methods to access eggs and nests; snakes invade the nest cavity, whereas crows and martens attack the nestlings from outside the nest entrance using their beaks or forelimbs^21^,^22^. I previously showed that seventeen-day-old nestlings (one day before the typical age of fledging), which respond to the two types of alarm calls with adaptive behaviours to avoid predation, jump out of the nest cavity in response to “jar” calls and crouch down inside the cavity in response to “chicka” calls^11^.

I hypothesized that incubating female Japanese great tits assess predation risk by deriving referential information from conspecific alarm calls given outside the nest cavity. I conducted a playback experiment that examined the response of incubating females to the two types of alarm calls in the absence of actual predators. Eighteen females bred in nestboxes were used for the experiment. After a female returned to her nestbox and sat on eggs, it was subjected to 1-min of “jar” calls, “chicka” calls, or background noise (control) that were played back from a speaker set up in front of the nestbox. I observed behavioural responses of females to playbacks by using a CCD camera attached to the ceiling of the nestboxes. All three treatments were tested in each female and the order of trials was counter-balanced.

## Results

**Figure 2:**
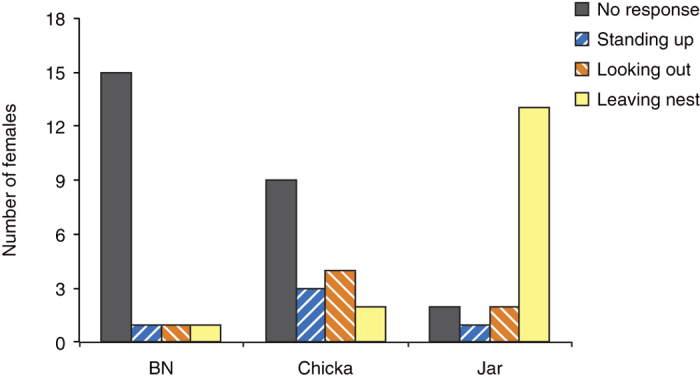
Responses of incubating female Japanese great tits (n = 18) to playbacks of “jar” calls, “chicka” calls, and background noise (BN).

Incubating female Japanese great tits responded differently to playbacks of “jar” calls, “chicka” calls, and background noise (Fig. 2). The response scores (see Methods) of females to playbacks varied across the three experimental treatments (CLMM, χ^2^ = 33.59, df = 2, P < 0.0001). In response to playback of “jar” calls, most females stepped onto the nest entrances (15/18), and many left the nestboxes (13/15) immediately after playback wa s commenced (median = 15 s, range: 1–33 s). In contrast, they rarely left the nestboxes during playbacks of “chicka” calls (2/18) and background noise (1/18). The response scores of females to “jar” calls were significantly higher than they were to “chicka” calls (post-hoc CLMM, χ^2^ = 16.34, df = 1, P < 0.0001, α = 0.025), and to background noise (post-hoc CLMM, χ^2^ = 46.58, df = 1, P < 0.0001, α = 0.017). Females always searched for and gazed at the ground near to the nestboxes after they left in response to playback of “jar” calls (13/13), but such behaviour was not observed when females left the nests during playbacks of “chicka” calls (n = 2) and background noise (n = 1). Females responded differently to “chicka” calls; three females stood on their nest materials and looked toward the nest entrances, four females stepped onto, and looked out of the nest entrances, and two females left the nestboxes. Nine females did not respond to playback of “chicka” calls. During playback of background noise, most of the tits stayed inside their nestboxes (17/18), rarely stood up (1/18), looked the outside (1/18), and left the nest (1/18). The response scores of females to “chicka” calls were significantly higher than they were to background noise (post-hoc CLMM, χ^2^ = 4.80, df = 1, P = 0.028, α = 0.05).

## Discussion

The different responses to two types of alarm calls would provide adaptive value for incubating females. The females left the nestboxes immediately after hearing playback of “jar” calls. Since snakes invade the nestboxes and may prey on adults as well as on eggs and nestlings^22^, escaping from nests before their invasion is the only way to avoid snake attack. After leaving the nests, all of the females gazed at the ground, a behaviour that would help them to detect snakes approaching the nest from the ground^20^. Once a snake is detected, females may approach it closely, spread out their wings and tails, and hover over it, which would help to disturb its invasion^22^. The tits looked out of the nest entrance in response to playback of “chicka” calls. This response may help the females gather information about the type and location of predators, since “chicka” calls are used for a variety of predator types, such as crows and martens^10^, that approach nests from different spatial locations (airs vs ground)^21^,^22^. Most females stayed in the nestboxes during playback of “chicka” calls; this might serve to prevent visually hunting predators from detecting the exact location of a nest by using parental activity near the nest^19^. Crows and martens cannot invade the nestboxes and may attack eggs from outside the nest entrances^21^,^22^. Nestlings of the tits often avoid predation by crows^22^ and martens^21^ by staying inside nestboxes. Therefore, incubating females may not be required to escape from the nest cavities when hearing “chicka” calls.

These results show that incubating female Japanese great tits derive information about predator type (i.e., snakes or non-snake predators) from conspecific alarm calls. Gathering information is crucial for animals to survive predation hazards and to defend their offspring^4^,^23^. Parents are limited by their visual fields when incubating eggs, particularly in nestboxes or in covered nests, and therefore the ability to gather information from auditory cues and signals that are associated with predators is required. It has been shown in other cavity-breeding or dome-nesting species that breeders sample predator information in the nest surroundings and react adaptively to risk outside the nest. For example, incubating female brown thornbills, *Acanthiza pusilla*, look out of their dome nests when hearing predators’ vocalizations, which may increase the amount of predator information gathered^24^. Incubating female house sparrows, *Passer domesticus*, use the sound made by an approaching predator to detect an approaching attack^25^. Alarm calls may provide more detailed information about predators (e.g., predator distance and behaviour) to incubating parents, as described in intra-group communication in several birds^26^,^27^ and mammals^28^,^29^,^30^. Therefore, further study is required to elucidate how finely incubating parents can extract information about predators from conspecific calls or other auditory cues from predators, and how they respond to the perceived risk.

Since the type of threat that causes breeding failure may vary during the breeding cycle of birds^3^, the function of alarm calls may be different between different breeding stages. For example, females of the yellow warbler respond to brood parasite specific alarm calls by nest protection behaviour, and this response is more intense at egg-laying stage than at nestling stage^15^. In the case of Japanese great tits, the response of parents to alarm calls is different between incubating and nestling periods. Whereas incubating females showed predator avoidance or information gathering behaviour in response to the two types of alarm calls, they respond to these calls by predator-searching behaviours during the nestling period; they gaze at the ground in response to “jar” calls (i.e., searching for snakes) and scan the horizon in response to “chicka” calls (i.e., searching for non-snake predators)^20^. Older nestlings also respond to the different alarm calls by appropriate predator avoidance behaviours^11^. The intensity of alarm calls varies throughout the breeding cycle: the calling rate is typically lower during incubation stage and increases as nestlings grow up^3^,^9^,^16^. While variation in the intensity of alarm calls has been explained in terms of parental investment theory^3^, it might also reflect changes in the intended targets of alarm calls as well as the type of risk that they face^16^,^31^.

The present findings may represent a novel example of functionally referential communication, which may shed new light on the evolution of communication^12^,^13^. Snakes pose a unique threat because they use specific hunting tactics in comparison to other predator types, which require a specific escape strategy for females and nestlings. Therefore, divergence of nest predator types may provide a selective force for the evolution of multiple types of alarm calls in breeding birds. In addition, nest architecture may also be an important ecological factor determining the complexity in communication. Incubating parents and nestlings of cavity-nesting species may have selected to use two contrasting behaviours to avoid snakes and other predators, i.e., leaving the nest or staying at the nest. In contrast, for open-nesting species, it would be sufficient for both incubating parents and nestlings to use the same escape strategy because leaving the nest is the best avoidance strategy for all predator types. Further comparative work with regard to predator diversity and nest architecture may help in our understanding of evolutionary pathway of functionally referential communication in animals.

## Methods

### Study site and subjects

I collected data from 15 May to 4 June 2014 on 18 female Japanese great tits inhabiting in a mixed deciduous–coniferous forest in Karuizawa, Nagano, Japan (36°21–22’N, 138°35–36’E). I attached nest boxes to tree trunks at 1.8 m from the ground, and the females used these boxes for their first broods of the season. The average clutch size was 9 ±1 (mean ± SD, n = 18). During nine breeding seasons from 2006 to 2014, up to 8% of females were depredated during incubation, and the predation rate on eggs ranged from 4% to 17% of nests (unpublished data).

### Playback preparation

I constructed playback stimuli from alarm calls that were previously recorded from Japanese great tits of the study population in the 2009 and 2010 breeding seasons. The “jar” calls were elicited by exposure to a live Japanese rat snake near the nestboxes, whereas the “chicka” calls were elicited using a stuffed jungle crow^10^,^11^. Whereas “jar” calls are specifically produced for snakes, “chicka” calls are used for predators other than snakes and their note composition subtly varies with the type of predator (e.g., crows vs martens)^10^. In this study, I only used the “chicka” calls given for crows in “chicka” call playbacks to control for the possibility that females respond differently to the subtle variation in these calls.

I used Adobe Audition 3.0 software (Adobe Corporation, San Jose, CA, U.S.A.) to construct the playback stimuli. I chose 90 “jar” calls from the recordings of nine individuals (five males and four females) and 90 “chicka” calls from the recordings of 10 individuals (six males and four females) on the basis of the sound quality (i.e., when the signaller was close to the microphone when it called and there was low background noise). Since tits seem not to discriminate between alarm calls of males and females^20^, calls from both sexes were used for the experiment. I chose five calls from the same individual and recorded them onto a sound file at a rate of 5 calls/12 s (roughly one call every 2.4 s), and repeated the 12-s file to fill a 1-min sound file. I filtered out low-frequency (<1 kHz) noise and amplified the calls to the same loudness level on a computer in order to equalize the amplitude across call playbacks. I created background noise files in the same way as the call files. Thus, I obtained 18 unique exemplars for each treatment. I saved all the sound files in WAV format (16-bit accuracy, 48.0 kHz sampling rate) on an SD memory card. I used unique exemplars for each focal female to avoid pseudoreplication^32^.

### Experimental procedure

I tested the response of female Japanese great tits (n = 18) to playbacks of “jar” calls, “chicka” calls, and background noise (control). An AT-SPG50 speaker (Audio-Technica Corporation, Tokyo, Japan) was attached atop a tripod at a height of 1.25 m from the ground and 2.0 m from the nest, and was directed toward the nestbox. The playback speaker was connected to an R-09HR digital audio recorder using EXC-12A extension cords (Victor Company of Japan, Kanagawa, Japan), which enabled the control of playbacks from an observation position 15 m away from the nest. I played back alarm calls at a constant volume (75 dB at 1 m from the speaker measured using an SM-325 sound level meter; AS ONE Corporation, Osaka, Japan), and background noise at the same amplitude as the background noise level of the call playbacks (50 dB at 1 m). All three stimuli (“jar” calls, “chicka” calls, and background noise) were tested for each female. Females were subject to playback of calls that were recorded from individuals other than their mates or neighbours to control for the effect of familiarity. Playbacks began at least 3 min after females returned to the nestbox, in the absence of birds calling outside of the nestbox. No males visited the nests during 1-min of playbacks. Each trial was separated by at least 1.5 h to reduce habituation. The order of playbacks was counter-balanced. Trials took place under calm and dry weather conditions between 08:30 and 16:00 h (Japan Standard Time).

I recorded female responses to alarm calls by using a KPC-EX20B CCD camera (KT&C Corporation, Seoul, Korea) connected to a GZ-MG880 digital video camera (Victor Corporation, Kanagawa, Japan). Behavioural responses of females during 1-min playbacks were categorized and scored as follows: (i) No response (0 points): females continued to incubate eggs and did not show any marked responses. (ii) Standing up (1 point): females stood up on their nest materials and looked at the nest entrances. (iii) Looking out of the nest entrance (2 points): females stood up on their nests, stepped on the nest entrances, and looked out of the nest entrances. (iv) Leaving the nest (3 points): females stepped on the nest entrances and left the nestboxes. I also recorded the time at which females left the nestboxes. When females left the nestboxes during the 1-min playbacks, I continued the observation of the behaviour of females until the playbacks were finished. I noted whether females gazed at the ground after leaving the nests because this response has been known as a specific behaviour to search for and detect snakes^20^.

### Statistical analysis

I used cumulative link mixed models (CLMMs) to analyse the effect of playback treatments on the female response scores. Primary analysis was conducted by including treatment (“jar”, “chicka”, and background noise) as a fixed term and individual identity of focal birds as a random term. In the event of a significant effect of treatment, I further performed post-hoc pairwise comparison between treatments (“jar” versus “chicka”, “chicka” versus background noise, and “jar” versus background noise). In the same way as the primary analysis, the models for the pairwise comparisons included treatment as a fixed term and individual identity of focal birds as a random term. CLMMs were run using R for Mac version 3.1.1^33^ with the *clmm* function in the package *ordinal*. I used two-tailed, log likelihood ratio tests to calculate P values. The level of significance was first set at α = 0.05, but the α value was adjusted according to the sequential Bonferroni method^34^ when making multiple comparisons.

### Ethical statement

All experiments were performed in accordance with relevant guidelines and regulations. All experimental protocols were approved by the Animal Care and Use Committees at the Graduate University for Advanced Studies and adhered to the Guidelines for the Use of Animals in Research of the Animal Behavior Society/Association for the Study of Animal Behaviour. This research was performed under permission from the Ministry of the Environment and the Forestry Agency of Japan.

## Additional Information

**How to cite this article**: Suzuki, T. N. Assessment of predation risk through referential communication in incubating birds. *Sci. Rep.* **5**, 10239; doi: 10.1038/srep10239 (2015).

## Figures and Tables

**Figure 1:**
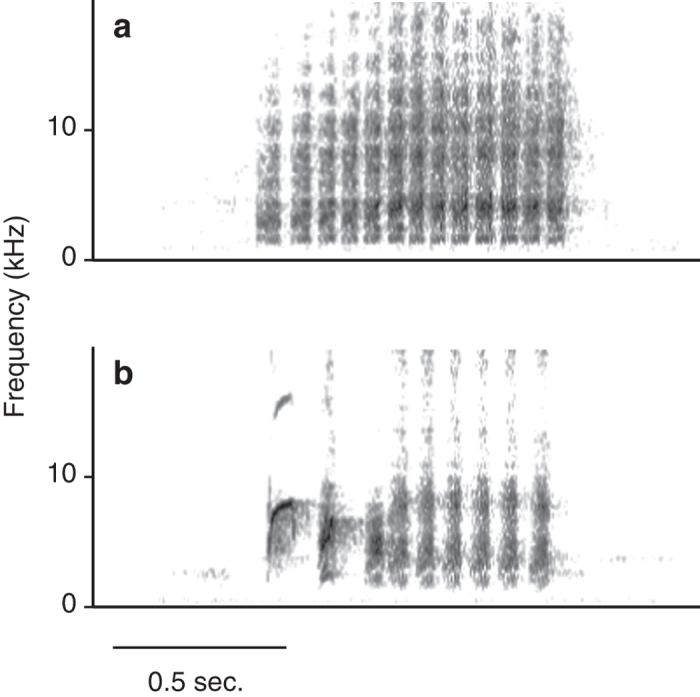
Sound spectrograms of two types of alarm calls: (**a**) “jar” and (**b**) “chicka” calls.
